# Offline to online: my musical odyssey to publication

**DOI:** 10.1038/s41467-023-44474-7

**Published:** 2024-01-03

## Abstract

As part of our tenth anniversary celebrations, the editorial team at Nature Communications wanted to hear from early career researchers who have published with us. We asked the early career researchers to tell us in an essay what is amazing about the research question(s) that drove them and the highs—and lows—of the journey from hypothesis to publication.

**Figure:**
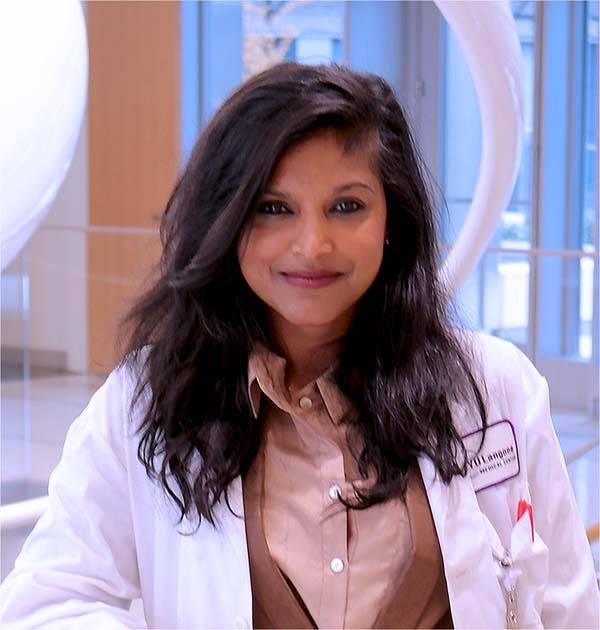
Bhama Ramkhelawon

Bhama Ramkhelawon is an Associate Professor in the Department of Surgery at New York University Langone Medical Center. Her research program is focussed on investigating the mechanisms that promote vascular diseases, such as aortic aneurysms and peripheral vascular disease. Why is this important? She believes that unlocking the inflammatory attributes that fuel these life-threatening pathologies might open much-needed promising therapeutic gateways to alleviate the burden of patients affected by these diseases.

Q: As an early career researcher and an author of papers published by Nature Communications^1,2^, tell us about the journey you’ve been on?

**A:** In high school, the heart murmurs “lub dub” were one of the exciting revelations of the marvels of the human body that guided my studies in the cardiovascular field. As a kid, I remember putting my head against the chest of my pets to make sure that the heart was beating, but, looking back now, I realize that I was contemplating to travel into the cardiac muscle and speak to the cells so they could tell me the tale of how they create the magic of the lub and the dub that sustain the beat of our lives. Was it too loud in there for them, I wondered? I used to live in such a quiet town at that time, that I could hear my own heart drums during silent nights. At bedtime, I used to pause, change the rhythm of my breathing, so I could focus on the altered acoustics. As I moved to busier and bigger cities, the echoes of my nocturnal heartbeats were absorbed by the noisy city activities. The voice of the mighty conductor was muted and the tubular pieces of the orchestra maintained the tempo of the symphony. At the same time, I grew a deep interest in finding out how the skeleton of the blood vessels is architected in such a way that they can never rest from the beating waves of the heart. I felt that these were very intelligently designed tubular structures of the body and I was going to get some answers into how they function so tirelessly. They are composed of the perfect mixture of elasticity and rigidity conferring leverage to simultaneously absorb the pulsating shocks, but also strengthen the infrastructure. Why are these vascular ramifications key instigators that determine the tempo of our body? And is the cardiovascular system the orchestra that animates the opera of our body?

A couple of decades later, I sit at my desk at New York University Langone Medical Center as a new principal investigator directing a team of scientists with the same questions roaming in my mind. I realized that this was a challenging task - the journey is going to be sparkled by excitement and deceptions, but these are definitely worth the voyage to the concert. I was also struck by the fact that we don’t fully understand how the healthy human body works, yet we have to investigate what occurs when tissues and organs of our body fail when faced with diseases, which are oftentimes life-threatening. Aortic aneurysm is one such disease that affects the aorta, degrades its structure, weakens its tubular framework, ruptures the tube, floods the body with uncontained blood, and is life-threatening in 90% of the ruptured cases. All of these events happen silently, rarely with a chirp of notice. Aortic aneurysms are a mysterious silent killer. An intruder that dares to disrupt abruptly the serenade of our body.

Why and how aneurysms affect the main artery that irrigates the body, as unwanted hooligans would, is one of the foci of my laboratory. Our initial quest was driven by the question of why the body is unable to sense the initial trauma and consequently arm itself with a defence system to combat such injuries. Naturally, we focused on investigating the immune component that nestles in the aorta. Taking advantage of single-cell RNA sequencing analysis of the aneurysmal aorta, we were able to detect that in this compromised condition, one of the forefront immune soldiers, macrophages, turn against smooth muscle cells that normally consolidate the arterial structure. We demonstrated that macrophages release an axon guidance molecule that is captured by neighbouring vascular smooth muscle cells expressing its receptor. This leads to reprogramming of the smooth muscle cells to release proteolytic enzymes that degrade the aortic layers and predispose to rupture. Alas, macrophages sang the wrong lyrics to the smooth muscle cells, thereby hijacking the sonata into a drama. In this work, we unveiled the complex multicellular dialogue that nourishes aneurysm disease development (Hadi et al. Nat Commun 9, 5022 (2018)). Whether we can re-tune the vocals of the macrophages, is one of the follow-up questions that we are currently addressing.

Still driven by the silent and melancholic vocals of aneurysms, we took a step back and looked at the bigger picture. We analyzed the co-morbidities and/or risk factors that co-exist with aneurysm pathology to gain insights into the maestro that could lead the dark acts of the vascular drama. We found that the snippet of solfege resonating from the damaged pulmonary organs reach the sensitive ears of the pirated macrophage soldiers patrolling the caves of the aorta. And again, the soldiers misbehave and in acts of conspiracy destroy its own habitat in the final act of the opera (Boytard et al. Nat Commun 11, 4311 (2020)). The original hypothesis to study the connection between the lungs and the aorta stemmed from our unique laboratory setting in the department of surgery, where we can work hand in hand with clinicians. This collaboration allowed us to demonstrate that modulating inflammation in the lung, increased proteolytic damage in the aorta and precipitated aneurysm development. We showed that this outcome was driven by the leakage of HMGB1 from damaged lung epithelia, which targets resident aortic macrophages leading to RIPK3-dependent activation of the proteolytic enzyme MMP12. Careful observation of microscopy images of RIPK3-deficient macrophages allowed us to decipher the critical role of mitochondria in this process. Now that we understand that macrophages residing in the aorta have attuned ears and resonate to distant cantors emanating from the lungs, we are investigating strategies to make macrophages deaf and/or mute pulmonary cells using CRISPR/Cas9 technology.

As with most original pieces, the reviewer critics were initially sceptical, but convincible by additional proofs. Some of the first comments were satirical and hurtful. As you can imagine, reading 5 pages of criticisms sometimes requesting impossible transgenic mice models (which by itself would almost guarantee a Nobel prize) or focusing on supplementary figure 25A instead of main figures 1 to 7 panel A to Z, raised questions about whether our beloved Mozart-like manuscript was being recategorized into an outdated boy band summer pop song. However, even in a boy band album, there are some songs that stick with us. In the same way, I appreciated some of the comments of the reviewers that allowed us to perform additional experiments to further support our findings, describing the pathological dialogue between the diseased lungs and aorta. I will leave it up to you to guess what the musical tastes of our beloved editors are - Mozart or boy band? In any case, receiving the email notifying us of our manuscript acceptance sounded like Beethoven was playing the fifth symphony just for my lab with the members of the boy band watching from the back…Our hope for the future is to fix the strings of the broken instruments and restore the symphony of the famous lub dub opera for individuals whose volume is unfortunately tuned down by aneurysm pathology. As you will appreciate, I am constantly inspired and draft most of our working hypotheses based on the dynamic behaviors found in music. What our research has shown is that diseases that affect the aorta often reflect polyphonic singing rather than solos. See you in the next acts of the modern aneurysmal Magic Flute from the Ramkhelawon lab. Stay tuned!
